# Peripheral T cell lymphoma following CD19-targeted chimeric antigen receptor T cell therapy

**DOI:** 10.1007/s12185-025-04074-1

**Published:** 2025-10-21

**Authors:** Hiro Tatetsu, Koji Kato, Atsushi Wada, Taichi Hirano, Shikiko Ueno, Yuko Miyasato, Asami Yamada, Takafumi Shichijo, Yusuke Higuchi, Yujiro Ueda, Kisato Nosaka, Yoshiki Mikami, Kennosuke Karube, Masao Matsuoka, Jun-ichirou Yasunaga

**Affiliations:** 1 Department of Hematology, Rheumatology, and Infectious Diseases, Kumamoto University School of Medicine, Kumamoto University Hospital, 1-1-1, Honjo, Chuo-ku, Kumamoto, 860-8556 Japan; 2 Department of Medicine and Biosystemic Science, Kyushu University Graduate School of Medicine, Fukuoka, Japan; 3 Department of Diagnostic Pathology, Kumamoto University Hospital, Kumamoto, Japan; 4 Department of Hematology and Oncology, Kumamoto Red Cross Hospital, Kumamoto, Japan; 5 Department of Pathology and Laboratory Medicine, Graduate School of Medicine, Nagoya University, Nagoya, Japan

**Keywords:** CAR T cell, PTCL, Secondary malignancies

## Abstract

Chimeric antigen receptor (CAR) T cell therapy has significantly improved outcomes for patients with refractory B cell lymphoma. However, rare cases of secondary T cell lymphomas have raised safety concerns. Here, we present a case of peripheral T cell lymphoma not otherwise specified (PTCL-NOS) that developed eight months following CD19-directed CAR T cell therapy (lisocabtagene maraleucel) in a 66-year-old male patient with recurrent diffuse large B cell lymphoma. The patient initially achieved complete remission but later developed a subcutaneous mass and systemic lymphadenopathy. Histopathology and flow cytometry confirmed a diagnosis of PTCL-NOS with a CD3 + , CD8 + , and CD30 + phenotype, as well as clonal T cell receptor gene rearrangements. No immunoglobulin rearrangements were detected, ruling out a lineage switch. Furthermore, the CAR transgene was undetectable by RNA-in situ hybridization, and flow cytometry showed no CAR protein expression, suggesting that the lymphoma was not caused by CAR gene integration. This case highlights the importance of re-biopsy in cases of suspected relapse following CAR T cell therapy, and emphasizes the need for long-term monitoring. While a direct causal link remains unclear, industry–academia collaboration is crucial for investigating the mechanisms underlying secondary T cell malignancies and improving the safety of CAR T cell therapy.

## Introduction

Chimeric antigen receptor (CAR) T cell therapy has shown promising results in treating refractory B cell lymphoma, with high response rates and durable remissions [1]. However, an analysis of the FDA Adverse Event Reporting System (FAERS) data found that among 12,394 adverse events, 536 (4.3%) were secondary primary malignancies, including cases of secondary T cell malignancies, raising serious safety concerns [2–4]. Gene therapy for X-linked severe combined immunodeficiency (SCID) has previously been associated with insertional oncogenesis-induced T cell lymphomas [5], raising concerns that CAR T cell therapy may carry similar risks.

As of December 31, 2023, the FDA had reported 22 cases of T cell malignancies among over 27,000 CAR T cell therapy recipients in the U.S., with CAR gene integration confirmed in three of those cases [3]. On January 19, 2024, the FDA issued a warning regarding the risk of T cell malignancies linked to CAR T cell therapies targeting CD19 or BCMA. Although the role of CAR gene integration remains unclear, reported cases are limited [6]. Here, we present a case of peripheral T cell lymphoma (PTCL) following CD19-targeted CAR T cell therapy.

## Patient’s clinical presentation

**Fig. 1:**
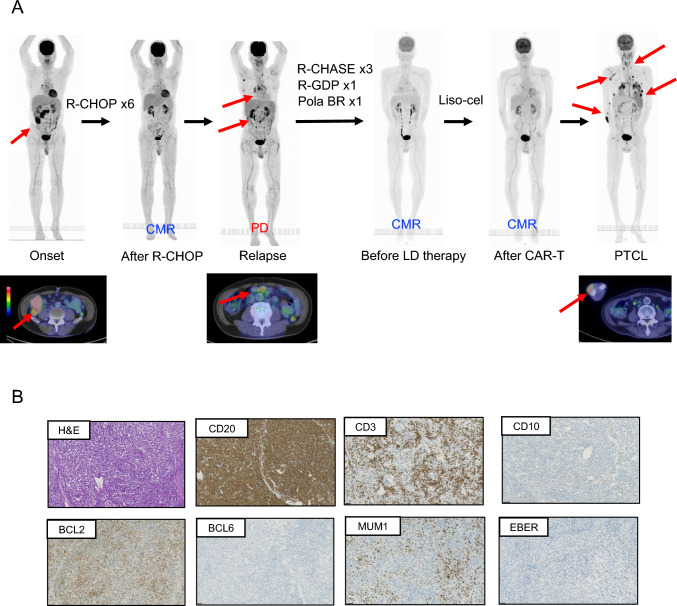
Clinical course and pathological findings of the lymph node at initial onset. **A** Clinical course and PET-CT images, **B** Pathological findings at the time of onset. The lymphocytes were positive for CD20, BCL-2 and MUM1, and negative for CD3, CD10 and BCL6. EBV-encoded small RNA-in situ hybridization (EBER-ISH) was negative. *PET-CT* positron emission tomography-computed tomography, *R-CHOP* rituximab, cyclophosphamide, doxorubicin, vincristine, and prednisolone, *CHASER* cyclophosphamide, high-dose cytarabine, dexamethasone, etoposide, and rituximab, *R-GDP* rituximab–gemcitabine, dexamethasone and cisplatin, *Pola-BR* rituximab, polatuzumab vedotin, and bendamustine, *LD* lymphodepleting, *Liso-cel* lisocabtagene maraleucel, *CAR* chimeric antigen receptor, *H&E* hematoxylin and eosin, *EBER-ISH* EBV-encoded small RNA-in situ hybridization

A 66 year-old man, with a history of diabetes mellitus, was found to have anemia and a nodular chest lesion in his chest during a routine check-up in July 2020. Positron emission tomography-computed tomography (PET-CT) in September 2020 showed mediastinal, abdominal, and mesenteric lymphadenopathy **(**Fig. 1A**)**. An open abdominal lymph node biopsy was performed, and a microscopic examination revealed a proliferation of large atypical lymphoid cells, which were positive for CD20 and BCL-2, partially positive for MUM1, and negative for CD3, CD10 and BCL6. EBV-encoded small RNA-in situ hybridization (EBER-ISH) was negative. These findings were indicative of a non-Germinal Center B-Cell (GCB) DLBCL **(**Fig. 1B**)**. Bone marrow biopsy showed no infiltration of lymphoma cells.

The patient received six cycles of rituximab, cyclophosphamide, doxorubicin, vincristine, and prednisolone (R-CHOP) achieving complete remission (CR) confirmed by PET-CT in May 2021 **(**Fig. 1A**)**. However, a follow-up PET-CT in May 2022 revealed a relapse of the disease. Cyclophosphamide, high-dose cytarabine, dexamethasone, etoposide, and rituximab (CHASER) therapy was started in June 2022. After three courses of CHASER therapy, the tumor showed no reduction in size; hence, the treatment was changed to rituximab–gemcitabine, dexamethasone, and cisplatin (R-GDP) therapy. Due to the refractory nature of the disease, the patient agreed to undergo CAR T cell therapy.

After the completion of the lymphocyte harvest, bridging therapy with rituximab, polatuzumab vedotin, and bendamustine (Pola-BR) was initiated in September 2022. Lymphocyte reduction therapy with fludarabine and cyclophosphamide, CAR T cells (Lisocabtagene Maraleucel; Liso-cel) were infused in December 2022. The patient suffered from COVID-19 post-infusion and was treated with nirmatrelvir/ritonavir and remdesivir. A complete metabolic response (CMR) was confirmed by PET/CT scan in March 2023 **(**Fig. 1A**)**.

**Fig. 2:**
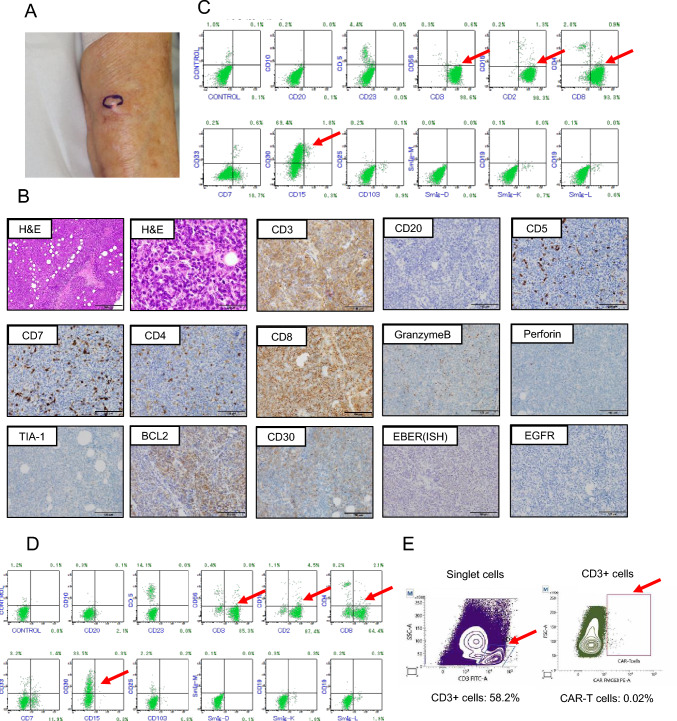
Pathological findings for the subcutaneous mass. **A** Photograph of a skin tumor, **B** Pathological findings on skin tumor. Immunohistochemical staining showed positive for CD3, BCL-2, and CD30, a predominance of CD8-positive cells. Granzyme B was positive in some areas, perforin was weakly positive in very few areas, and TIA-1 was negative. CD20, PAX5, CD79a, and EGFR were negative. EBER-ISH was also negative, **C** Flow cytometry analysis on skin tumor, **D** Flow cytometry analysis on bone marrow sample, **E** Flow cytometric analysis for CAR T cells. *H&E* hematoxylin and eosin, *EBER-ISH* EBV-encoded small RNA-in situ hybridization

In August 2023, a subcutaneous mass was found on the right forearm during routine follow-up. PET-CT showed multiple enlarged lymph nodes, spleen infiltration, and a subcutaneous mass, suggesting a relapse of DLBCL **(**Fig. 1A**)**. A biopsy of the subcutaneous mass was performed **(**Fig. 2A**)**, and the microscopic examination revealed a diffused proliferation of medium- to large-sized atypical lymphoid cells with pleomorphic nuclei with coarse nuclear chromatin texture. Immunohistochemically, these atypical lymphoid cells were positive for CD3, BCL-2, CD8, and CD30, whereas CD5, CD7, CD20, PAX5, CD79a, cytotoxic molecules, and EGFR were negative. EBER-ISH was also negative, hence leading to the diagnosis of peripheral T cell lymphoma (PTCL‐NOS) **(**Fig. 2B**)**. Flow cytometry analysis showed that tumor cells were positive for CD3, CD8, and CD30 **(**Fig. 2C**)**. The bone marrow examination showed lymphomatous involvement showing features similar to those of the subcutaneous lesions in flow cytometry **(**Fig. 2D**)**.

PCR results showed gene rearrangement of the T cell receptor Vβ/Jβ2, Dβ/Jβ, and Vγ/Jγ, while no gene rearrangement of immunoglobulin was observed in bone marrow samples, suggesting that PTCL cells were derived from a T cell clone although lineage switch from DLBCL cells was unlikely. To evaluate the presence of the CAR transgene in PTCL-NOS cells, RNA-in situ hybridization (ISH) for CAR transgene in the FFPE sample was performed and showed negative results (Bristol Myers Squibb). The number of tumor cells in the peripheral blood gradually increased. Flow cytometry was performed on the peripheral blood sample at the 55% tumor cell stage to detect CAR. The results suggested that the tumor was negative for CAR protein, indicating that it was not a PTCL caused by CAR gene integration **(**Fig. 2E**)**. The patient’s condition deteriorated progressively, and palliative care was required.

## Discussion

CAR T cell therapies targeting CD19 or BCMA poses a risk for T cell malignancies, including CAR-positive lymphomas. T cell lymphoma was previously reported in two patients in a phase I trial using piggyBac transposons for non-viral CD19 CAR T cell therapy [7, 8]. Both cases exhibited high transgene copy numbers and genomic alterations. Another case involved a patient treated with ciltacabtagene autoleucel (cilta-cel) for multiple myeloma who later developed CAR-positive T cell lymphoma [9]. Despite achieving CMR after CHOEP-21, the patient eventually required hematopoietic stem cell transplantation. Additionally, a patient receiving cilta-cel developed an asymptomatic CAR-positive CD4 + cytotoxic T cell lymphoma [10].

Transient CAR-integrated T cell proliferation has been previously reported in some studies. A chronic lymphocytic leukemia patient in a clinical trial for CD19 CAR T cell therapy (trial: NCT01029366) exhibited clonal CAR T cell expansion due to retroviral insertion disrupting the *TET2* gene [11]. Similarly, a patient with acute lymphoblastic leukemia experienced clonal CAR T cell expansion due to retroviral insertion into the CBL-B locus, without malignant transformation [12].

The incidence of T cell lymphoma following CAR T cell therapy is estimated to be 0.06% (22/34,400 cases) [4]. A meta-analysis of four studies (1253 participants) reported secondary cancer incidence to be similar between CAR T cell therapy (5%) and standard treatment (4.9%) groups [13]. Genetic mutations, including *DNMT3A* and *TET2*, involved in clonal hematopoiesis, have been identified in both DLBCL and secondary T cell lymphoma [14].

The current case under discussion underscores the importance of histological confirmation by re-biopsy in suspected relapses after CAR T cell therapy. Our analyses using RNA ISH and flow cytometry did not detect CAR expression; however, these techniques may not be sensitive enough to detect low-level or transcriptionally silent CAR transgene insertions. On comparing these techniques to more sensitive techniques, such as digital droplet PCR and integration-site mapping, as described in recent reports [10, 15], the absence of CAR detected in our case does not definitively rule out the presence of integrated CAR sequences. This limitation should be considered when interpreting the results of diagnostic evaluations of secondary lymphomas following CAR T cell therapy.

Furthermore, collaborative research between academia and industry is essential to elucidate the mechanisms underlying secondary T cell lymphomas. While academia can contribute real-world clinical data and access to tissue samples, industry retains proprietary resources, such as CAR vector sequences, detection reagents, manufacturing processes, and safety databases. Sharing these resources is critical for accurately identifying and investigating potential CAR-related oncogenic events, particularly in rare secondary malignancies, such as T cell lymphoma. Long-term studies are mandatory to improve the safety of CAR T cell therapies and to address the risks of secondary malignancies.

## Data Availability

Inquiries for data should be directed to tatetsu@kumamoto-u.ac.jp. The data will be made available to achieve the aims of the approved proposal.
